# Weighted-Attribute Triplet Hashing for Large-Scale Similar Judicial Case Matching

**DOI:** 10.1155/2021/6650962

**Published:** 2021-04-16

**Authors:** Jiamin Li, Xingbo Liu, Xiushan Nie, Lele Ma, Peng Li, Kai Zhang, Yilong Yin

**Affiliations:** ^1^School of Software, Shandong University, Jinan, China; ^2^School of Computer Science and Technology, Shandong Jianzhu University, Jinan, China; ^3^Shandong Liju Robot Technology Co., Ltd, Yantai, China

## Abstract

Similar judicial case matching aims to enable an accurate selection of a judicial document that is most similar to the target document from multiple candidates. The core of similar judicial case matching is to calculate the similarity between two fact case documents. Owing to similar judicial case matching techniques, legal professionals can promptly find and judge similar cases in a candidate set. These techniques can also benefit the development of judicial systems. However, the document of judicial cases not only is long in length but also has a certain degree of structural complexity. Meanwhile, a variety of judicial cases are also increasing rapidly; thus, it is difficult to find the document most similar to the target document in a large corpus. In this study, we present a novel similar judicial case matching model, which obtains the weight of judicial feature attributes based on hash learning and realizes fast similar matching by using a binary code. The proposed model extracts the judicial feature attributes vector using the bidirectional encoder representations from transformers (BERT) model and subsequently obtains the weighted judicial feature attributes through learning the hash function. We further impose triplet constraints to ensure that the similarity of judicial case data is well preserved when projected into the Hamming space. Comprehensive experimental results on public datasets show that the proposed method is superior in the task of similar judicial case matching and is suitable for large-scale similar judicial case matching.

## 1. Introduction

Owing to the rapid development of the social economy, the number of judicial cases handled by courts shows a trend of steady increase yearly, and people in the judicial field will endure substantial pressure at work. Among the cases handled by the courts, most were similar. In actual work, handling both common and rare cases requires considerable manpower and material resources. Luckily, algorithms such as machine learning and natural language processing have entered the stage of rapid development and are being broadly applied in our daily life scenarios. It is imperative to integrate artificial intelligence technology into case analysis and trial processes. An important subtask in smart justice is the task of similar judicial case matching. Similar judicial case matching tasks can provide the most similar cases when legal professionals are handling cases, to realize consistent judgement results of similar cases and promote the process of judicial fairness and justice.

Similar judicial case matching is essentially used to determine the similarity of documents. There are previous studies that focused on determining the similarity among documents of different cases. Raghav et al. [1] transformed the document into embeddings using the vector space model and measured the similarity between two embeddings. Wu et al. [2] used Word2Vec to train the word vectors of legal documents to form the document vector space model and found the most similar top *N* legal documents from the text matrix. Mueller et al. [3] proposed a twin-network model based on LSTM for calculating semantic similarity. However, when the scale of the judicial case document data to be matched is large, the computational cost of traversing the document database will also be large; therefore, the abovementioned method is not suitable for large-scale similar judicial case matching scenarios. In recent years, hashing, a representative method of approximate nearest neighbor search, has garnered widespread attention owing to its low storage and high computational efficiency.

As the most well-known approximate nearest neighbor search method, hashing can map high-dimensional data to compact binary codes in the Hamming space. It has garnered considerable interest in recent years owing to its great efficiency gains in massive data [4–6]. The distance between binary hash codes is measured using the Hamming distance, which can be quickly solved using XOR operations. Therefore, the hash method has great advantages in terms of storage and efficiency [7, 8]. However, similar judicial case documents are always clustered together in the feature space, and the hash codes mapped from them have a high collision probability, making it difficult to accurately identify them. Therefore, in response to existing problems, the method proposed in this paper introduces the triple loss function to reduce the intraclass distance and increase the interclass distance, making the finally learned hash code more discriminative and improving the accuracy of matching similar judicial documents. At the same time, the hashing method can greatly increase the retrieval speed owing to its binary representation and has great value in improving the efficiency of large-scale judicial case data matching [9].

Similar judicial case matching mainly compares the text similarity of the two judicial documents and selects the most similar document from the two candidate documents, which is essentially the text similarity task. However, owing to the general description framework and the professional words in judicial documents [10], higher requirements are presented for the matching method of similar judicial cases. In comparison with the common text similarity computing task, a similar judicial case matching task faces more challenges. First, judicial case documents are written in a fixed format containing a large number of legal terms, and there are several common words in the factual description part; thus, the mechanism through which to preprocess the judicial document is very important. Second, the length of the judicial documents is quite long, and it is difficult for machines to interpret a long description of facts. Furthermore, the semantic representation is also very complicated. Third, the differences between case documents can be subtle, making it difficult to determine whether the two documents are similar. It can be seen that the feature attributes in the judicial case documents become the key to determining the difference between similar cases. However, efficiently obtaining the appropriate weight of each attribute in judicial case documents remains an open issue.

To overcome the aforementioned limitations, we propose a novel triple deep hashing method, namely, weighted-attribute triplet hashing (WATH), to facilitate similar judicial case matching. Considering the structural complexity of judicial cases document, the proposed method obtains the weight of judicial feature attributes based on hash learning. The binary code of each document is then used to match similar judicial cases in a large corpus. Our method can benefit the similar judicial case matching in the judicial domain to help the people in the legal field work better and promote the process of judicial fairness and justice. The contributions of this study can be summarized as follows:

  This study proposes a similar judicial case matching method based on triple deep hash learning, which performs fast similarity matching by converting judicial case documents into hash codes. Specifically, this method establishes a loss function term based on the similarity of the triplet document, generates the hash code for similarity matching, and improves the matching efficiency.
  The weight of each judicial feature attribute is learned using a deep neural network such that the important attributes will get adaptive weights during the hash codes learning procedure.
  The experimental results show that the proposed method improves the speed and accuracy of similar judicial case matching and is suitable for large-scale similar judicial case matching.

The remainder of this paper is organized as follows. We briefly introduce related work on similar judicial case matching and representative hashing methods in Section 2.  Section 3 presents our proposed approach.  Section 4 provides the experimental results on the public dataset. Finally, the conclusions are presented in Section 5.

## 2. Related Work

In this section, we review related works from the following two aspects: similar judicial case matching and hash learning.

### 2.1. Similar Judicial Case Matching

Similar judicial case matching aims to calculate the similarity between case facts, which is essentially the calculation of text similarity. Similar judicial case matching is a significant practical application of text matching, which is a considerable issue in natural language processing. Currently, there are several models for text similarity calculation in academia. TF-IDF is a statistical method that is often used for text classification and information retrieval. Generally, it only considers the number of documents and the frequency of keywords appearing in documents and requires a lot of training data. The Word2Vec [11] model is a popular text modeling method. It parses legal documents into a collection of words, uses language models to model the contextual relationship between words, and maps words to an abstract low-dimensional real-valued space to generate corresponding word vectors. The bidirectional encoder representations from transformer (BERT) [12] model can be used as a universal language representation model. Its goal is to use a large-scale unlabeled corpus to train and obtain features of text containing rich semantic information.

To date, traditional text matching has been extensively studied, and there are several classic approaches in the field of text matching. Currently, studies on similar judicial case matching are mostly conducted on the basis of text similarity tasks, and the method is optimized according to the specific situation of the task. Song et al. [13] used the bus fire incident as an example and established a case similarity matching model by combining the information weight and text similarity calculation methods. Liu et al. [14] established a text similarity model and algorithm for medical dispute cases based on the knowledge of the doctor-patient field. Most studies on text similarity focus on text similarity in general fields, and there are a few text similarity calculation methods in the judicial field. Kumar et al. [15] analyzed the problem of finding similar legal judgements by extending the similarity measure used in information retrieval and web documents. Thenmozhi et al. [16] extracted lexical features from all the legal documents and the similarity between each current case document, and all the prior cases documents are determined using cosine similarity scores. Saravanan et al. [17] compared the extracted features from legal case documents rather than the full texts.

### 2.2. Hash Learning

Existing learning-based hashing methods can be generally divided into two categories as follows: unsupervised hashing and supervised hashing. Unsupervised hashing methods attempt to construct a hash function on the training data points to preserve the similarity between the original space and Hamming space. Some well-known unsupervised hashing methods include spectral hashing (SH) [18, 19], iterative quantization (ITQ) [20], anchor graph hashing (AGH) [21], discrete graph hashing (DGH) [22], and K-means hashing (KMH) [23]. However, unsupervised hashing methods do not use semantic information. Thus, long codes are required to further improve the search accuracy. Supervised hashing is employed to design the hash function using label information [24, 25]. Representative supervised hashing methods include minimal loss hashing (MLH) [26], supervised hashing with kernels (KSH) [27], fast supervised discrete hashing (FSDH) [28], supervised short-length hashing (SSLH) [29], and supervised discrete hashing with mutual linear regression (SDHMLR) [30].

However, traditional hash functions do not work well for large datasets. Owing to the development of deep learning, deep hashing methods [31–34] achieve higher performance than traditional methods. The design of the loss function is an important part of the deep supervised hashing method. The intuition of the loss function design is to maintain similarity [35]. According to the similarity preserving method, the deep hashing algorithms can be generally divided into pairwise similarity preserving and multiwise similarity preserving. Representative deep hashing methods based on pairwise similarity preserving include deep supervised hashing [36], deep pairwise-supervised hashing [37], deep hashing network [38], and deep asymmetric pairwise hashing [39]. Triplet loss is the most popular loss in the category of deep hashing algorithms which use the multiwise similarity preserving loss function. Representative deep hashing methods based on triplet loss include bit-scalable deep hashing [40], one-stage deep hashing [41], and triplet loss hashing [42]. To handle multiview features, some efforts [43–45] and [46] have been made towards effective cross-view hashing.

## 3. Proposed Method

In this section, the proposed WATH method is introduced in detail. We first provide the definition of the problem addressed in this work. Thereafter, we introduce the mechanism through which we can learn the function and obtain the weight of the case feature attributes. Finally, we show the triplet loss function of our model.

### 3.1. Notation and Problem definition

Without loss of generality, we focus on similar judicial case matching for private lending data. Similar judicial case matching aims to calculate the similarity between case documents. We take a triplet (*I*, *I*^+^, *I*^−^)_*i*=1_^*n*^ as the input, where *I*, *I*^+^, and *I*^−^ are fact descriptions of three judicial cases. Case *I* is the anchor sample, case *I*^+^ is a positive sample, and case *I*^−^ is a negative sample. The aim of hash learning for texts is to learn a mapping *F* : *I*⟶{0,1}^*q*^, such that an input text *I* can be encoded into *q*-bit binary code *F*(*I*). Our goal is to measure case *I*^+^ and case *I*^−^ with *I* and predict the case that is more similar to *I*.

### 3.2. Model Architecture

**Figure 1:**
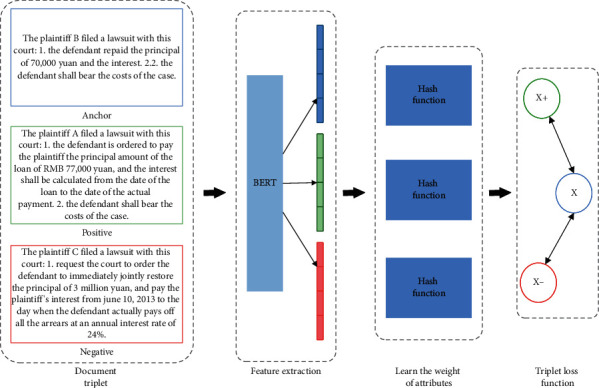
Framework of the proposed approach.

In this paper, we propose an architecture of triplet deep hashing designed for judicial similar case matching, as shown in Figure 1. It first represents the characteristics of judicial case documents by BERT. Subsequently, the attributes of the case documents are divided into four parts. Next, the attribute weights are learned to train the hash functions. In addition, judicial case documents are converted into hash codes for fast similarity matching. In this section, we present the details of these parts.

#### 3.2.1. Attribute Feature Extraction Module

Judicial case documents are usually written in a structured format containing many legal terms, and there are several common words in the factual description part; thus, it is hard to select the document most similar to the target document in a large corpus [47]. In addition, the differences between judicial case documents can be subtle, which means that similar judicial case matching is more challenging than normal text matching in information retrieval. It can be seen that the attribute feature in the judicial case documents becomes the key to determine the difference between similar cases. To solve these problems, determining the weight of each attribute of a judicial document becomes fundamental. In comparison with the traditional text-matching model, the method proposed in this paper solves the problem in which different attributes are of equal importance to the matching decision.

Before the documents are sent into the hash function, WATH extracts the judicial feature attributes and converts them into the feature vector using the BERT model. The language model BERT [12] is the state-of-the-art model released by Google. It aims to utilize the bidirectional attention mechanism and huge unsupervised corpora to output effective context-sensitive representations of each word in a sentence. In contrast to encoding using term vectors such as Word2Vec and ELMo [48] in the representation layer of the classical natural language model, the BERT model is a pretraining model that can effectively obtain the long-range dependence existing in the document; thus, it can realize encoding of relations between sentences in a similar judicial case text.

We extract a set of specific judicial feature attributes according to the structure and representation of judicial documents. Generally, similar judicial causes will have the same feature attributes in judicial documents. The crucial attributes in private lending for each document include the following: (1) the description of plaintiffs and defendants, including their property and the count of plaintiffs and defendants, (2) the claims of the plaintiff, including the facts and reasons described and the evidence presented by the plaintiff, (3) the defendant's defense, including whether the defendant accepted the fact of the loan and the evidence presented by the defendant, and (4) the statement of the court's decision, including the relationship between the plaintiff and defendant, amount borrowed, and repayment situation.

#### 3.2.2. Learning Hash Function Based on Attribute Weight

**Figure 2:**
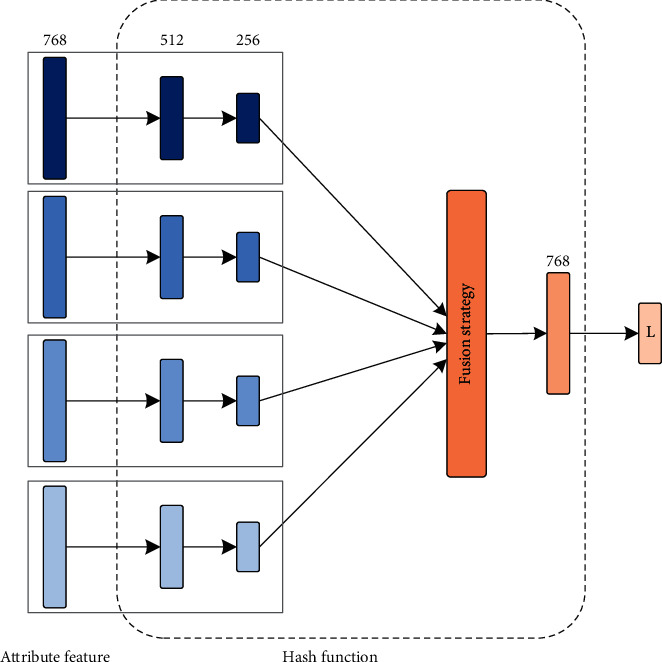
Learning hash function based on attribute weight.

After obtaining document attribute feature vectors from the BERT, we propose a deep network module to project these document features into compact hash codes based on weighted attributes. We suppose that each target hash code has *L* bits. As can be seen in Figure 2, the proposed hash function module first assigns different weights to the input four parts attribute features. Here, the hash function learns the weighted feature attributes of each case document as follows:

(1) $$\begin{array}{c} {\left\{g_{i}\left(x_{i}\right)\right\}}_{i=1}^{4}, \end{array}$$

where *x*_*i*_ ∈ *R*^256^ are the feature attribute vectors of each document generated by the BERT model and *g*_*i*_(*·*) is the deep neural network to obtain the weight of different feature attributes.

After the weight of the feature attributes is obtained, we need to combine this weighted feature vector. We use the following two fusion strategies to combine the weighted feature vectors: concatenation and elementwise addition.

(a) Concatenation: the concatenation method directly combines these attributes feature vectors to a superfeature vector so that the input vector can be re-expressed as *g*_1_(*x*_1_), *g*_2_(*x*_2_), *g*_3_(*x*_3_), and *g*_4_(*x*_4_). Then, the concatenated vector is

(2) $$\begin{array}{c} V_{\text{con}}=\left[g_{1}\left(x_{1}\right);g_{2}\left(x_{2}\right);g_{3}\left(x_{3}\right);g_{4}\left(x_{4}\right)\right]. \end{array}$$

We can see that the dimensionality of the concatenation method combined attribute feature vector is 256 × 4.

(b) Elementwise addition: another strategy to fuse attribute feature vectors is a parallel combination method. Rather than the concatenation combination method, which combines different attribute feature vectors into a supervector, the parallel combination method combines attribute feature vectors through elementwise addition. The formulas are expressed as follows:

(3) $$\begin{array}{c} V_{\text{add}}=g_{1}\left(x_{1}\right)+g_{2}\left(x_{2}\right)+g_{3}\left(x_{3}\right)+g_{4}\left(x_{4}\right), \end{array}$$

where + is the elementwise product between two vectors. The dimensionality of the elementwise addition combined attribute feature vector is still 256. The elementwise addition strategy can enhance the importance of judicial case semantics information.

#### 3.2.3. Loss Function Module

**Figure 3:**
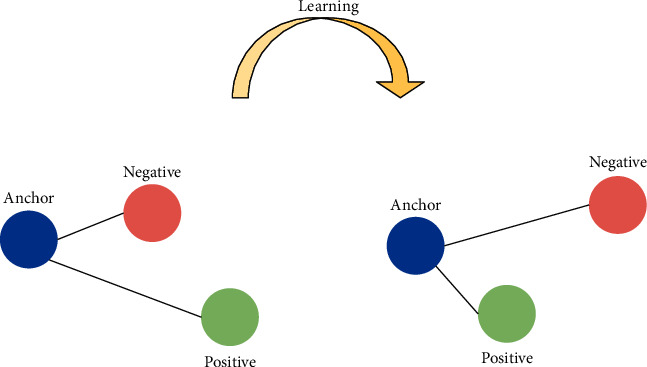
Diagram of triple relationship.

In the existing supervised hashing method, the auxiliary information is mostly in the shape of pairwise labels to indicate that the semantics of the sample pairs are similar or dissimilar. However, a pairwise label only ensures that one constraint is observed. Triplet loss was first proposed in FaceNet by Google to train face embeddings for the recognition task [49]. As demonstrated in Figure 3, triplet loss uses a set of training samples, including an anchor, positive, and negative samples. In particular, triplet loss aims to ensure that the distance between the anchor and positive samples becomes smaller through training and learning, whereas the distance between the anchor and negative samples becomes larger. Therefore, the triplet loss is introduced in this paper.

Specifically, for a given form of instrument sample triplet (*I*, *I*^+^, *I*^−^), the document *I*^+^ is more similar to the query document *I* than the document *I*^−^. Such triplet ranking information, i.e., sim(*I*, *I*^+^) > sim(*I*, *I*^−^) is expected to be encoded in the learned binary hash codes. After fusing the four weighted feature attributes, we produce a unified vector representation *V* of the document. The goal of the method in this study is to learn a mapping *F* that makes the binary hash code *F*(*V*) closer to *F*(*V*^+^) not *F*(*V*^−^). The loss function based on triples can be expressed as follows:

(4) $$\begin{array}{c} L_{\text{triplet}}\left(F\left(V\right),F\left(V^{+}\right),F\left(V^{-}\right)\right)=\max\left(0,{\left\|F\left(V\right)-F\left(V^{+}\right)\right\|}_{2}-{\left\|F\left(V\right)-F\left(V^{-}\right)\right\|}_{2}+\alpha \right),\\ \text{s}.\text{t}.\;F\left(V\right),F\left(V^{+}\right),F\left(V^{-}\right)\in {\left[0,1\right]}^{L}, \end{array}$$

where *α* is a threshold parameter that measures the distance between matched and unmatched document pairs. In this study, multiple thresholds are used to conduct experiments, and *α* is set to 0.5. For a given triplet, this loss function can maximize the distance between the matched document and the unmatched document pair.

### 3.3. Out-of-Sample Extension

**Figure 4:**
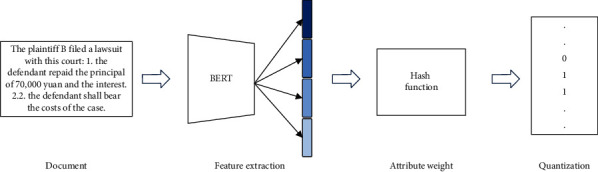
Process of generating hash code for an out-of-sample.

After the network has been trained, we still need to obtain the hashing codes of the case documents that are not in the training data. As shown in Figure 4, for an unseen document triplet, we obtain its binary code by inputting it into the WATH network. Specifically, according to the structure information, an unseen judicial case document is first divided into four parts, and then, we extract the attribute feature as the document representation using BERT. Subsequently, the weights of the feature attributes are obtained using a hash function. In addition, the attribute feature vector of each judicial case document is fused according to different fusion strategies. Finally, judicial case documents are converted into hash codes for fast similarity matching.

### 3.4. Computational Complexity

The computational complexity of WATH is composed of three parts: extracting document features, learning binary code, and calculating Hamming distance between documents. The computational complexity of extracting document features is BERT model complexity. The computational complexity of learning binary code is *O*(*nm*^*k*^), where *m* depends on the width of neural networks and *k* depends on the depth of neural networks. The computational complexity of calculation Hamming distance between documents is *O*(1).

## 4. Experiments

In this section, we evaluate the match performance of WATH on the public dataset using several representative hashing methods. We first introduce the details of the dataset, evaluation criteria, comparison methods, and implementation details. Thereafter, experimental results and discussions are provided to make fair comparisons. Finally, the convergence of WATH is further investigated.

### 4.1. Dataset

We conduct experiments on a public dataset from the China Judgements Online website^1^. This dataset contains 5,000 triplets of judicial case documents, which are related to Private Lending. Every document in the triplets contains the fact description part. The documents are presented in triples, whose members are three case documents: one query document *A* and two documents *B* and *C* to be matched. We ensure that the positive sample *B* is closer to the query document *A* than the negative sample *C*; that is, sim(*A*, *B*) > sim(*A*, *C*).

### 4.2. Evaluation Metric

The precision score is used to evaluate the match performance. The precision score measures whether the matched data belong to the same class as the query. The larger the precision score, the better the match performance. The precision score is defined as follows:

(5) $$\begin{array}{c} \text{Precision}=\frac{\text{TP}+\text{TN}}{\text{ALL}}, \end{array}$$

where TP denotes the number of correctly matched data, TN the number of correctly unmatched data, and ALL the total number of samples.

### 4.3. Baseline Methods

The proposed WATH model is compared with the following five representative hashing methods.

  SH [18] first introduces the spectral analysis into the hashing technique, which tries to find the data-dependent compact hash codes such that the neighboring data points in the original Euclidean space are still neighbors in the Hamming space [50].
  PCA-ITQ [20] aims to maximize the data variance of each bit via PCA projection and reduces the quantization error between the rotated PCA-embedded data and the discrete codes.
  PCA-RR [20] attempts to minimize the quantization error by randomly rotating the datapoints and makes the variance more balanced.
  MFH [51] preserves the local structure for each individual feature by considering the local structures of all the features in a global fashion [52].

### 4.4. Implementation Details

We use the following two different strategies for attribute feature fusion: concatenation and elementwise addition. The concatenation method directly combines these attribute feature vectors to a super feature vector. Another fusion strategy to fuse attribute features is a parallel combination method, which combines attribute feature vectors through elementwise addition.

To further search the role of feature attributes, we designed ablation experiments to evaluate the performance of the method in this study. This is the same as our proposed method, except for the module of weighting feature attributes. The learning rate is set as 10^−4^. The batch size and epochs are 128 and 130, respectively. The entire model was optimized using Adam. Specifically, Adam utilizes the first-order momentum *m*_*t*_ to retain the direction of the historical gradient. Meanwhile, the second-order momentum *v*_*t*_ is used to make the learning rate adaptive for Adam [53]. The Adam update equation can be expressed as follows:

(6) $$\begin{array}{c} \left\{\begin{array}{c} m_{t}=\beta_{1}m_{t-1}+\left(1-\beta_{1}\right)g_{t},\\ v_{t}=\beta_{2}v_{t-1}+\left(1-\beta_{2}\right)g_{t}^{2},\\ {\hat{m}}_{t}=\frac{m_{t}}{1-\beta_{1}^{t}},{\hat{v}}_{t}=\frac{v_{t}}{1-\beta_{2}^{t}},\\ W_{t+1}=W_{t}-\frac{\eta }{\sqrt{{\hat{v}}_{t}}+\varepsilon }{\hat{m}}_{t}, \end{array}\right. \end{array}$$

where *W*_*t*_ denotes the *t*^*th*^ iteration. *β*_1_ and *β*_2_ are two hyperparameters that are set to 0.9 and 0.999, respectively. ${\hat{m}}_{t}$ and ${\hat{v}}_{t}$ are the bias-current for the first and second moments, respectively. *ϵ* is usually set to 1*e* − 8, to avoid division by zero.

### 4.5. Results and Analysis

Table 1 shows the precision scores when we vary the number of hashing bits in {64,96,128,256,512,768} on the dataset. It is noteworthy that all the methods generally achieve higher precision scores with an increase in the hash code length. This is reasonable because longer hash codes can encode more semantic information about the document. Nevertheless, the highest precision scores are not always the longest. In particular, we can observe that the proposed WATH approach achieves comparable match performances in different hash length settings and significantly outperforms most baselines, *i.e.*, SH [18], PCA-ITQ [20], PCA-RR [20], and MFH [51]. The results verify the validity of the attribute weighted and triplet loss. In addition, the Pro_WS method removes the weighted feature attribute vectors, and the accuracy averagely decreases by 2.7%. Thus, we can see that feature attributes play a significant role in the proposed method.

**Figure 5:**
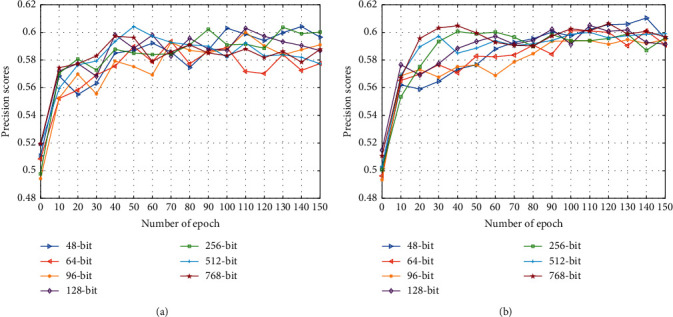
Precision score with different lengths of hash codes when the number of epochs increases. (a) The results of the utilization of the concatenation fusion strategy. (b) The results of the utilization of the elementwise addition fusion strategy.

Figure 5 shows the change in the precision score curves with the increase in iterations. The elementwise addition fusion strategy works better than the concatenation fusion strategy because the latter can enhance the importance of judicial case semantics information.

Table 2 shows the results of matching time comparing hash codes with real-valued representation. We investigated the computational time of our proposed methods and compared them with two real-valued methods. The matching time corresponds to the time of matching similar case documents in a triplet sample and does not include the feature extraction and the feature attribute vectors weighted. From Table 2, we have the following two observations. First, we can see that the matching time of our proposed hashing method with different hash code lengths is always faster than real-valued representation methods. When we set the code length to 128 bits, the matching speed of the hashing method is approximately 180 and 395 times faster than that of the real value method by using Euclidean distance and Cosine distance, respectively. Second, the storage cost of the real-valued method is 767 times larger than the 128 bit hash code. This is reasonable because each dimension of a binary code can be stored using only 1 bit, whereas several bytes are typically required for one dimension of the real value, leading to a dramatic reduction in storage cost. These results validate the effectiveness of WATH on large-scale datasets.

**Figure 6:**
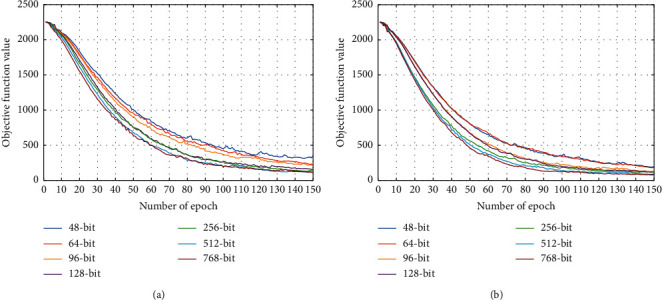
Convergence curves of the proposed method under hash codes with different lengths. (a) The fusion strategy of concatenation. (b) The fusion strategy of elementwise addition.

Figure 6 shows the convergence curves of WATH on the public dataset. We can draw three observations from Figure 6. First, we can observe that the proposed method converges rapidly, usually within 120 iterations of the dataset. The fast convergence speed indicates that the computational costs for learning hash codes are not expensive. Second, it can be observed that the objective function value generally presents a declining trend with the increase in epochs. As the number of epochs increases, the objective function value will eventually remain at a low level for different bits and tends to be stable. Finally, we note that the fitting of our model is better when the size of the bits increases. This implies that the proposed method will more easily distinguish the similarity of samples with an increase in the bit size. These results demonstrate that the proposed WATH has good convergence properties.

## 5. Conclusions

In this paper, we focus on the problem of similar judicial case matching in the judicial case domain and propose the WATH method to solve it. Considering that the judicial case attributes have logical connections, the model divides case documents into four parts according to structure information and then extracts the feature attributes using BERT. One of the main contributions of this paper is that it obtains the weight of different judicial feature attribute vectors by learning a hash function, and we use two fusion strategies to combine the weighted feature attribute vectors. We also introduced triplet constraints to ensure that judicial case data similarity is well preserved when projected into a Hamming space, and case documents are converted into hash codes for fast similarity matching. Comprehensive experimental results on the public dataset and convergence analysis have demonstrated the effectiveness of our algorithm, and the extracted judicial feature attributes are important for large-scale similar judicial case matching. This shows that our proposed method can benefit the similar judicial case matching in the judicial domain to help the people in the legal field work better. In the future, we aim to integrate a variety of loss functions to reduce the reconstruction loss [54].

## Figures and Tables

**Table 1:** Performance in terms of precision scores with deep different lengths of hash code.

Method	Hash bits						
	48 bits	64 bits	96 bits	128 bits	256 bits	512 bits	768 bits
SH	0.4750	0.4820	0.4850	0.4760	0.5050	0.5120	0.5200
PCA-ITQ	0.5066	0.5096	0.5106	0.5196	0.5040	0.5132	0.5160
PCA-RR	0.5114	0.5126	0.5070	0.5186	0.5098	0.5048	0.5074
MFH	0.5244	0.5206	0.5230	0.5258	0.5240	0.5230	0.5322
WATH_WS	0.5790	0.5630	0.5590	0.5800	0.5870	0.5620	0.5690
WATH_Con	0.5988	0.5717	0.6000	0.6030	0.5915	0.5925	0.5880
WATH_Add	0.6060	0.5904	0.5948	0.6018	0.5988	0.5976	0.5988

**Table 2:** Comparison of matching time (second) using hash codes and real-valued representation.

Method	Hash codes						Real value	
	64 bits	96 bits	128 bits	256 bits	512 bits	768 bits	Euclidean	Cosine
10^−5^ s	0.1698	0.1756	0.1964	0.3016	0.4272	0.6136	35.509	77.600

## Data Availability

Some or all data, models, or code generated or used during the study are available in a repository or online in accordance with funder data retention policies (http://wenshu.court.gov.cn/).
